# Functional Characterization of Core Regulatory Genes Involved in Sporulation of the Nematophagous Fungus Purpureocillium lavendulum

**DOI:** 10.1128/mSphere.00932-20

**Published:** 2020-10-28

**Authors:** Mi Chen, He-Yu Yang, Yan-Ru Cao, Quan-Quan Hui, Hai-Feng Fan, Chen-Chen Zhang, Jing-Jing Han, Zhi-Yi Guo, Jianping Xu, Ke-Qin Zhang, Lian-Ming Liang

**Affiliations:** a State Key Laboratory for Conservation and Utilization of Bio-Resources in Yunnan, Yunnan University, Kunming, China; b The Key Laboratory for Southwest Microbial Diversity of the Ministry of Education, Yunnan University, Kunming, China; c Key Laboratory of Special Biological Resource Development and Utilization of Universities in Yunnan Province, Department of Life Science and Technology, Kunming University, Kunming, China; d Department of Biology, McMaster University, Hamilton, Ontario, Canada; University of Georgia

**Keywords:** *Purpureocillium lavendulum*, asexuality, development, nematophagous fungi, conidiation, conidiophore

## Abstract

The nematophagous fungus Purpureocillium lavendulum is a natural enemy of plant-parasitic nematodes, which cause severe economic losses in agriculture worldwide. The production of asexual spores (conidia) in *P. lavendulum* is crucial for its biocontrol activity against nematodes. In this study, we characterized the core regulatory genes involved in conidiation of *P. lavendulum* at the molecular level. The central regulatory pathway is composed of three genes, P. lavendulum *brlA* (*PlbrlA*), *PlabaA*, and *PlwetA*, which regulate the early, middle, and late stages of asexual development, respectively. The deletion of *PlbrlA* completely inhibited conidiation, with only conidiophore stalks produced. PlAbaA determines the differentiation of conidia from phialides. The deletion of *PlwetA* affected many phenotypes related to conidial maturation, including abscission of conidia from conidium strings, thickening of the cell wall layers, vacuole generation inside the cytoplasm, production of trehalose, tolerance to heat shock, etc. Comparative analyses showed that the upstream regulators of the core regulatory pathway of conidiation, especially the “fluffy” genes, were different from those in *Aspergillus*. Besides their roles in conidiation, the central regulators also influence the production of secondary metabolites, such as the leucinostatins, in *P. lavendulum*. Our study revealed a set of essential genes controlling conidiation in *P. lavendulum* and provided a framework for further molecular genetic studies on fungus-nematode interactions and for the biocontrol of plant-parasitic nematodes.

**IMPORTANCE** Plant-parasitic nematodes cause serious damage to crops throughout the world. *Purpureocillium lavendulum* is a nematophagous fungus which is a natural enemy of nematodes and a potential biocontrol agent against plant-parasitic nematodes. The conidia play an important role during infection of nematodes. In this study, we identified and characterized genes involved in regulating asexual development of *P. lavendulum*. We found that these genes not only regulate conidiation but also influence secondary-metabolite production. This work provides a basis for future studies of fungus-nematode interactions and nematode biocontrol.

## INTRODUCTION

Plant-parasitic nematodes cause serious damage to more than 3,000 known species of plants, including most agricultural crops, resulting in economic losses amounting to US $157 billion annually worldwide (1). Nematophagous fungi are a diverse group of fungal species capable of capturing or infecting nematodes. These fungi represent an important source of potential biocontrol agents against plant-parasitic nematodes. Purpureocillium lavendulum is a new fungal species discovered recently and a close relative of the nematophagous fungus Purpureocillium lilacinum, which has been the most widely used species for controlling nematodes (2–4). An obvious difference between the two species is that *P. lavendulum* cannot grow at above 35°C and is thus not considered an infectious threat to humans, while *P. lilacinum* can grow well at above 35°C (4). The lack of growth at above 35°C makes *P. lavendulum* a safer candidate as a biocontrol agent than *P. lilacinum*.

Because fungal infection of nematodes starts with conidia, the number of asexual spores (conidia) produced by fungal strains can directly affect the infectivity of a nematophagous fungus. Furthermore, the production of a large number of conidia is essential for the successful application of the biocontrol agent in agricultural fields. Therefore, understanding the regulatory mechanism of sporulation of *P. lavendulum* could help us enhance spore production and its utilization efficiency. At present, almost nothing is known about the molecular mechanism of sporulation in *P. lavendulum*. In this study, we investigated the function of several genes in sporulation of *P. lavendulum*. Orthologs of these genes are known to be involved in sporulation in the model filamentous fungus Aspergillus nidulans (5–7).

## RESULTS

### Characterization of the regulatory genes involved in sporulation of *P. lavendulum*.

BLASTp searches were carried out to identify the putative genes involved in regulating sporulation in *P. lavendulum*. The queried genes included 11 genes total, including the 3 central regulators P. lavendulum *brlA* (*PlbrlA*), *abaA*, and *wetA*, the 6 “fluffy” genes *fluG* and *flbA* to -*E*, and *fadA* and *pkaA* (two inhibitors of conidiation in A. nidulans) as revealed in previous studies. We identified the orthologs of nine genes in the genome of *P. lavendulum*, including *PlbrlA*, *PlabaA*, *PlwetA*, *PlfadA*, and *PlpkaA* and the fluffy genes *PlfluG*, *PlflbA*, *PlflbC*, and *PlflbD*. However, the orthologous genes of *flbB* and *flbE* were not found in the *P. lavendulum* genome. The genes *PlbrlA*, *PlabaA*, *PlwetA*, *PlfadA*, and *PlpkaA* encode proteins of 367, 915, 614, 353, and 545 amino acids (aa), respectively. They showed 36%, 42%, 70%, 94%, and 80% amino acid sequence identity to their orthologs in A. nidulans. The four fluffy genes *PlfluG*, *PlflbA*, *PlflbC*, *PlflbD* encode proteins of 450, 705, 357, and 448 aa, respectively. They showed 31%, 59%, 47%, and 51% amino acid sequence identities to their orthologs in A. nidulans.

**FIG 1:**
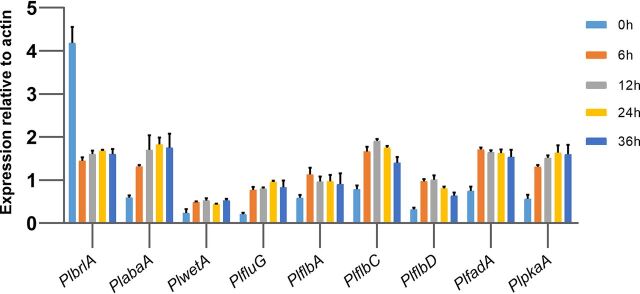
RT-qPCR data showing mRNA expression levels of nine conidiation-related genes in the *ku80* KO strain after various periods of conidiation induction.

Real-time quantitative PCR (RT-qPCR) was carried out to evaluate the expression profiles of the above-mentioned genes during conidiation in the fungus. Conidia of the *ku80* knockout (KO) strain were first inoculated in submerged liquid minimal medium (MM) (300 ml of medium in 500-ml flasks) and incubated stationarily at 28°C for 48 h. The mycelia were then collected by filtration and spread on top of solid MM to induce conidium production. Samples were taken at 0, 6, 12, 24, and 36 h after the start of incubation for RT-qPCR analysis. As shown in Fig. 1, *PlbrlA* showed a high expression level after 48 h of submerged cultivation and then dropped down during growth on solid media. This result is consistent with the feature of this fungus which can produce simple conidiophores from both aerial and submerged mycelia (4). The genes *PlabaA*, *PlwetA*, *PlfluG*, *PlflbA*, *PlfadA*, and *PlpkaA* showed regularly elevated expression during growth on solid media. *PlflbC* and *PlflbD* showed elevated expression during the first 12 h and then dropped down in the subsequent 24 h of cultivation.

### Roles of identified homologs in the regulation of conidiogenesis in *P. lavendulum*.

To elucidate the functions of the putative homologs identified as described above, we constructed deletion mutants for each of the genes in *P. lavendulum*, via homologous recombination. Here, the open reading frame (ORF) of each gene was replaced with the chlorimuron resistance gene (*sur*) and the replacements were confirmed by PCR and Southern blotting (Fig. S1 to S8). We constructed the complementing *PlwetA* plasmid using the In-Fusion methodology with a benomyl resistance gene (*bml*) as the selection marker; the details of the complementation of Δ*PlwetA* are described in Fig. S2.

10.1128/mSphere.00932-20.1 FIG S1 Knockout of the *PlabaA* gene in *P. lavendulum*. (A) The disruption plasmid of *PlabaA* and the relative position of *PlabaA* in the *ku80* KO strain. The relative positions of primers used for the construction of knockout plasmids and the verification of transformants are indicated. The relative position of restriction endonuclease sites and the probe used in Southern blotting are also shown. “Sur” represents the chlorimuron resistance gene. (B) Confirmation of the disruption of *PlabaA* by PCR in the mutants with chlorimuron resistance was achieved with primer pair sur5/sur3. −, the wild-type strain; +, the knockout plasmid; M, DNA marker. (C) Confirmation of the disruption of *PlabaA* by PCR in the mutants with chlorimuron resistance was achieved with primer pair abaA-PlKu80-F/abaA-PlKu80-R. +, the wild-type strain; −, the knockout plasmid; M, DNA marker. (D) The transformants were confirmed to have no additional copy of the knockout cassette randomly inserted into the genome, by PCR with primers GFP-f/GFP-r. −, the wild-type strain; +, the knockout plasmid. (E) Southern blot analysis of *ku80* KO and mutant strains. The probe was PCR amplified with the genomic DNA of the *ku80* KO strain; the primers used are shown in Table S1. Download FIG S1, TIF file, 1.0 MB. Copyright © 2020 Chen et al. 2020 Chen et al. This content is distributed under the terms of the Creative Commons Attribution 4.0 International license.

10.1128/mSphere.00932-20.2 FIG S2 Knockout of the *PlwetA* gene in *P. lavendulum*. (A) The disruption plasmid of *PlwetA* and the relative position of *PlwetA* in the *ku80* KO strain. The relative positions of primers used for the construction of knockout plasmids and the verification of transformants are indicated. The relative position of restriction endonuclease sites and the probe used in Southern blotting are also shown. Sur, the chlorimuron resistance gene. (B) Confirmation of the disruption of *PlwetA* by PCR in the mutants with chlorimuron resistance was achieved with primer pair wetA-5f/wetA-3f. −, the wild-type strain; +, the knockout plasmid; M, DNA marker. (C) The transformants were confirmed to have no additional copy of the knockout cassette randomly inserted into the genome, by PCR with primers GFP-f/GFP-r. −, the wild-type strain; +, the knockout plasmid. (D) Southern blot analysis of the *ku80* KO strain, *PlwetA* mutant, and complementary strain. The probe was PCR amplified with the genomic DNA of the *ku80* KO strain; the primers used are shown in Table S1. Download FIG S2, TIF file, 0.5 MB. Copyright © 2020 Chen et al. 2020 Chen et al. This content is distributed under the terms of the Creative Commons Attribution 4.0 International license.

**FIG 2:**
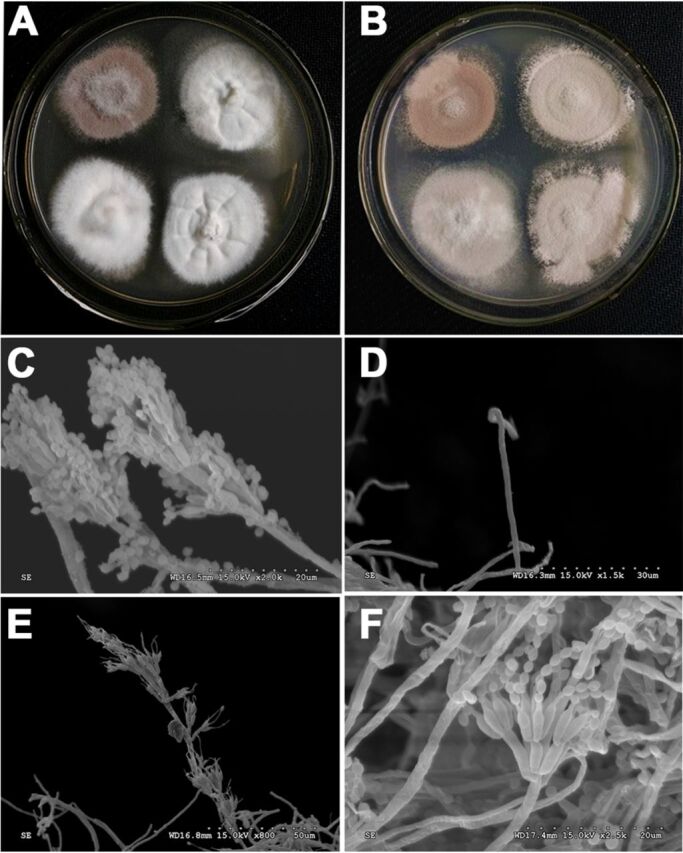
Morphological features of the strains with knockouts of *PlbrlA*, *PlabaA*, and *PlwetA* in *P. lavendulum*. (A) Colonies of the *ku80* KO strain (top left) and three Δ*PlabaA* strains; (B) colonies of the *ku80* strain (top left) and three Δ*PlwetA* strains; (C) SEM of the *ku80* KO strain asexual conidiogenous structure; (D) SEM of a stalk-like structure of the Δ*PlbrlA* strain; (E) SEM of the Δ*PlabaA* mutant asexual conidiogenous structure; (F) SEM of the Δ*PlwetA* asexual conidiogenous structure.

**FIG 3:**
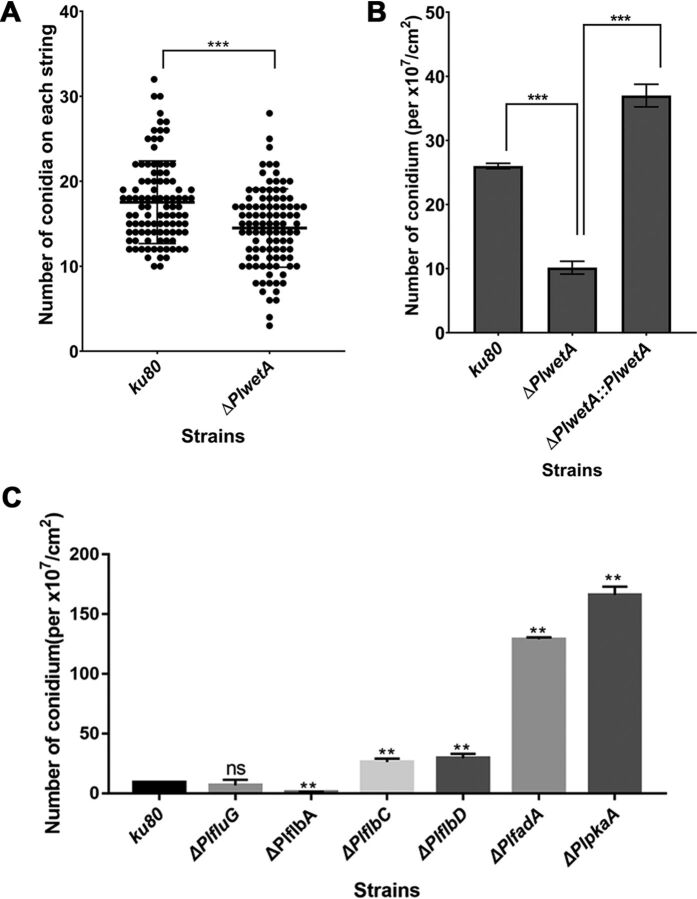
Statistical comparisons of conidia produced by the *ku80* and other knockout strains of *P. lavendulum*. (A) Comparison of conidial numbers on conidial chains between the *ku80* KO and Δ*PlwetA* strains. (B) Comparison of conidia produced between the *ku80* KO and Δ*PlwetA* strains and the complemented strains. (C) Conidia produced by the *ku80* and other knockout strains. **, *P* < 0.01; ***, *P* < 0.001; ns, not significant.

The morphological comparisons between the mutants and wild-type strain were conducted through light microscopy. The colonies of the *ku80* KO strain were purple, while the colonies of the Δ*PlabaA* and Δ*PlwetA* strains were white and off-white, respectively (Fig. 2A and B). Our previous study showed that the *PlbrlA* mutant also had white colonies (8). Using a scanning electron microscope (SEM), it was observed that the *ku80* KO strain developed normal conidiophores (Fig. 2C). The *PlbrlA* mutant produced no sporulation structure aside from having a stalk (Fig. 2D). The deletion of *PlabaA* resulted in immature phialides and extremely elongated hyphae with abnormal morphology (Fig. 2E). These results indicate that *PlbrlA* and *PlabaA* are essential for the differentiation of phialides in *P. lavendulum*. The *PlwetA* mutant had complete phialide structures, with chains of conidia longer than for the *ku80* KO strain (Fig. 2F). To confirm this phenotype, we cultured these strains on sterile microscope slides (see Materials and Methods) and then counted the number of spores on each spore chain directly under a light microscope. As shown in Fig. 3A, for selection of 100 spore chains of each strain randomly, we found that the length of spore chains of the *ΔPlwetA* strain were shorter than those of the *ku80* KO strain. Meanwhile, when the *PlwetA* null mutant was inoculated on solid medium, it exhibited reduced and limited conidiation when counted after the conidia were washed from the medium (Fig. 3B). This result revealed that the reduced conidium production in the Δ*PlwetA* strain could be due to fewer differentiated conidia from the phialides as well as the defect in conidium maturation, which could cause fewer conidia to be released from the phialides.

The putative fluffy genes identified through homology searches also showed significant effects on conidium production in *P. lavendulum*. When the fluffy gene *PlfluG* was knocked out, the mycelial growth rate increased slightly and the production of conidia was delayed (Fig. S8E). Interestingly, the final total count of conidia was not affected (Fig. 3C). The Δ*PlflbA* strain showed off-white colonies and produced a few conidia (Fig. 3C), and the whole colony of the Δ*PlflbA* strain began to autolysis after 20 days of cultivation, with only a small number of vegetative mycelia remaining. The growth rates of the colonies of both the Δ*PlflbC* and Δ*PlflbD* mutants were lower than that of the *ku80* KO strain, but they both produced more conidia than the *ku80* KO strain (Fig. 3C). When the *PlfadA* and *PlpkaA* genes were knocked out, the mutants produced more conidia than the *ku80* KO strain, indicating that these genes negatively regulate conidium production (Fig. 3C).

10.1128/mSphere.00932-20.3 FIG S3 Knockout of the *PlfadA* gene in *P. lavendulum*. (A) The disruption plasmid of *PlfadA* and the relative position of *PlfadA* in the *ku80* KO strain. The relative positions of primers used for the construction of knockout plasmids and the verification of transformants are indicated. The relative position of restriction endonuclease sites and the probe used in Southern blotting are also shown. (B) Confirmation of the disruption of *PlfadA* by PCR in the mutants with chlorimuron resistance was achieved with primer pair fadA-5f/fadA-3f. −, the wild-type strain; +, the knockout plasmid; M, DNA marker. (C) The transformants were confirmed to have no additional copy of the knockout cassette randomly inserted into the genome, by PCR with primers GFP-f/GFP-r. −, the wild-type strain; +, the knockout plasmid. (D) Southern blot analysis of the *ku80* KO and mutant strains. The probe was PCR amplified with the genomic DNA of the *ku80* KO strain; the primers used are shown in Table S1. Download FIG S3, TIF file, 0.6 MB. Copyright © 2020 Chen et al. 2020 Chen et al. This content is distributed under the terms of the Creative Commons Attribution 4.0 International license.

10.1128/mSphere.00932-20.4 FIG S4 Knockout of *PlpkaA* gene in *P. lavendulum*. (A) The disruption plasmid of *PlpkaA* and the relative position of *PlpkaA* in the *ku80* KO strain. The relative positions of primers used for the construction of knockout plasmids and the verification of transformants are indicated. The relative position of restriction endonuclease sites and the probe used in Southern blotting are also shown. (B) Confirmation of the disruption of *PlpkaA* by PCR in the mutants with chlorimuron resistance was achieved with primer pair sur5/sur3. −, the wild-type strain; +, the knockout plasmid; M, DNA marker. (C) Confirmation of the disruption of *PlpkaA* by PCR in the mutants with chlorimuron resistance was achieved with primer pair pkaA-PlKu80-F/pkaA-PlKu80-R. +, the wild-type strain; −, the knockout plasmid; M, DNA marker. (D) The transformants were confirmed to have no additional copy of the knockout cassette randomly inserted into the genome, by PCR with primers GFP-f/GFP-r. −, the wild-type strain; +, the knockout plasmid. (E) Southern blot analysis of *ku80* KO and mutant strains. The probe was PCR amplified with the genomic DNA of the *ku80* KO strain; the primers used are shown in Table S1. Download FIG S4, TIF file, 1.1 MB. Copyright © 2020 Chen et al. 2020 Chen et al. This content is distributed under the terms of the Creative Commons Attribution 4.0 International license.

10.1128/mSphere.00932-20.5 FIG S5 Knockout of the *PlflbA* gene in *P. lavendulum*. (A) The disruption plasmid of *PlflbA* and the relative position of *PlflbA* in the *ku80* KO strain. The relative positions of primers used for the construction of knockout plasmids and the verification of transformants are indicated. The relative position of restriction endonuclease sites and the probe used in Southern blotting are also shown. (B) Confirmation of the disruption of *PlflbA* by PCR in the mutants with chlorimuron resistance was achieved with primer pair flbA-f/flbA-r. −, the wild-type strain; +, the knockout plasmid; M, DNA marker. (C) The transformants were confirmed to have no additional copy of the knockout cassette randomly inserted into the genome, by PCR with primers GFP-f/GFP-r. −, the wild-type strain; +, the knockout plasmid. (D) Southern blot analysis of *ku80* KO and mutant strains. The probe was PCR amplified with the genomic DNA of the *ku80* KO strain; the primers used are shown in Table S1. Download FIG S5, TIF file, 0.5 MB. Copyright © 2020 Chen et al. 2020 Chen et al. This content is distributed under the terms of the Creative Commons Attribution 4.0 International license.

10.1128/mSphere.00932-20.6 FIG S6 Knockout of the *PlflbC* gene in *P. lavendulum*. (A) The disruption plasmid of *PlflbC* and the relative position of *PlflbC* in the *ku80* KO strain. The relative positions of primers used for the construction of knockout plasmids and the verification of transformants are indicated. The relative position of restriction endonuclease sites and the probe used in Southern blotting are also shown. (B) Confirmation of the disruption of *PlflbC* by PCR in the mutants with chlorimuron resistance was achieved with primer pair flbC-f/flbC-r. −, the wild-type strain; +, the knockout plasmid; M, DNA marker. (C) The transformants were confirmed to have no additional copy of the knockout cassette randomly inserted into the genome, by PCR with primers GFP-f/GFP-r. −, the wild-type strain; +, the knockout plasmid. (D) Southern blot analysis of *ku80* KO and mutant strains. The probe was PCR amplified with the genomic DNA of the ku80 strain; the primers used are shown in Table S1. Download FIG S6, TIF file, 0.4 MB. Copyright © 2020 Chen et al. 2020 Chen et al. This content is distributed under the terms of the Creative Commons Attribution 4.0 International license.

10.1128/mSphere.00932-20.7 FIG S7 Knockout of the *PlflbD* gene in *P. lavendulum*. (A) The disruption plasmid of *PlflbD* and the relative position of *PlflbD* in the *ku80* KO strain. The relative positions of primers used for the construction of knockout plasmids and the verification of transformants are indicated. The relative position of restriction endonuclease sites and the probe used in Southern blotting are also shown. (B) Confirmation of the disruption of *PlflbD* by PCR in the mutants with chlorimuron resistance was achieved with primer pair flbD-f/flbD-r. −, the wild-type strain; +, the knockout plasmid; M, DNA marker. (C) The transformants were confirmed to have no additional copy of the knockout cassette randomly inserted into the genome, by PCR with primers GFP-f/GFP-r. −, the wild-type strain; +, the knockout plasmid. (D) Southern blot analysis of *ku80* KO and mutant strains. The probe was PCR amplified with the genomic DNA of the *ku80* KO strain; the primers used are shown in Table S1. Download FIG S7, TIF file, 0.5 MB. Copyright © 2020 Chen et al. 2020 Chen et al. This content is distributed under the terms of the Creative Commons Attribution 4.0 International license.

10.1128/mSphere.00932-20.8 FIG S8 Knockout of the *PlfluG* gene in *P. lavendulum*. (A) The disruption plasmid of *PlfluG* and the relative position of *PlfluG* in the *ku80* KO strain. The relative positions of primers used for the construction of knockout plasmids and the verification of transformants are indicated. The relative position of restriction endonuclease sites and the probe used in Southern blotting are also shown. (B) Confirmation of the disruption of *PlfluG* by PCR in the mutants with chlorimuron resistance was achieved with primer pair fluG-f/fluG-r. −, the wild-type strain; +, the knockout plasmid; M, DNA marker. (C) The transformants were confirmed to have no additional copy of the knockout cassette randomly inserted into the genome, by PCR with primers GFP-f/GFP-r. −, the wild-type strain; +, the knockout plasmid. (D) Southern blot analysis of *ku80* KO and mutant strains. The probe was PCR amplified with the genomic DNA of the *ku80* KO strain; the primers used are shown in Table S1. (E) The colonies of *ku80* KO and Δ*PlfluG* strains in different media and at different times. Note that on day 7, the Δ*PlfluG* colonies were whiter than the *ku80* KO colonies, suggesting a delay of conidium production. Download FIG S8, TIF file, 1.7 MB. Copyright © 2020 Chen et al. 2020 Chen et al. This content is distributed under the terms of the Creative Commons Attribution 4.0 International license.

10.1128/mSphere.00932-20.9 FIG S9 The germination of *ku80* KO and Δ*PlwetA* strains in liquid MM. Bar = 10 μm. Download FIG S9, TIF file, 2.3 MB. Copyright © 2020 Chen et al. 2020 Chen et al. This content is distributed under the terms of the Creative Commons Attribution 4.0 International license.

### Deletion of *PlwetA* caused defective conidia.

The *wetA* gene in A. nidulans encodes a protein required for the synthesis of conidium-specific cell wall layers in the final stages of conidium maturation (9). We wondered whether the absence of *PlwetA* might affect maturation of conidia in *P. lavendulum*.

**FIG 4:**
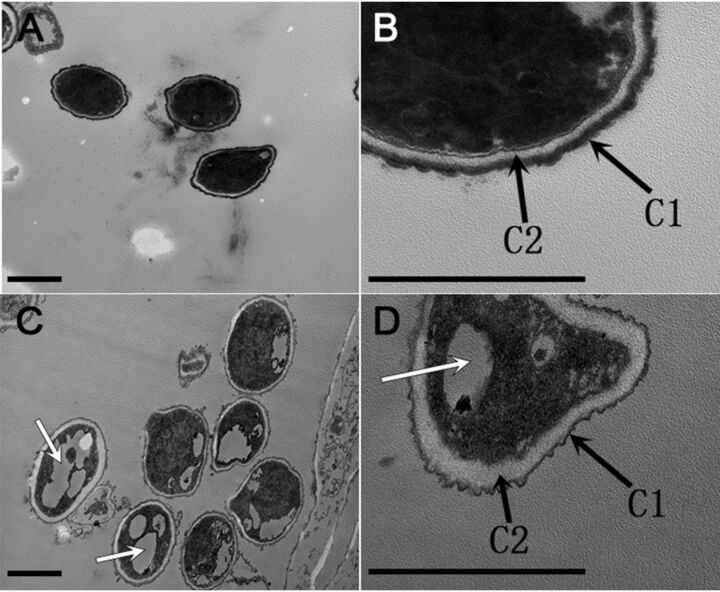
Transmission electron micrographs of conidia of *ku80* KO (A and B) and Δ*PlwetA* (C and D) strains of *P. lavendulum*. The white arrows in panel C indicate vacuoles in conidia. Black arrows indicate the C1 and C2 layers of the conidial cell wall, respectively. Bar = 1 μm.

To investigate whether *PlwetA* knockout would change the cell structure of conidia, we obtained transmission electron micrographs (TEM) of the cell wall structure and subcellular components of the *ku80* KO strain and the Δ*PlwetA* mutant. Conidia from the *ku80* KO strain formed an electron-dense C1 layer and a condensed electron-light C2 layer (Fig. 4A and B). However, in the Δ*PlwetA* strain, although both the C1 and C2 layers were formed, they both failed to condense, leading to a somewhat looser and thicker cell wall than in the wild-type strain (Fig. 4C and D). The cytoplasm of conidia from the Δ*PlwetA* mutant consisted mainly of vacuoles, accounting for approximately 64% of all conidia, while such vacuoles were found in less than 5% of the conidia of the *ku80* KO strain (Fig. 4C and D). These observations implied that *PlwetA* plays an essential role in conidial cell wall integrity and conidial maturation.

**FIG 5:**
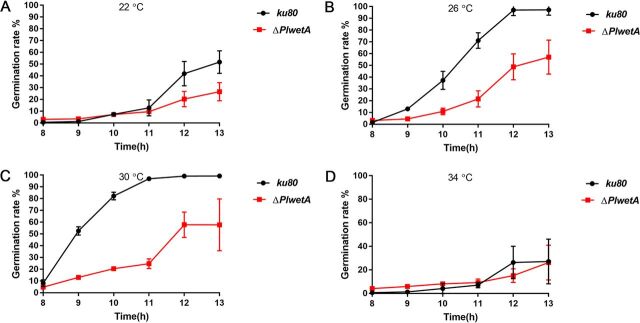
Germination rate comparison between *ku80* KO and Δ*PlwetA* strains of *P. lavendulum* at different temperatures.

To further evaluate conidium maturation in the Δ*PlwetA* strain, we compared the germination ratios of conidia of the Δ*PlwetA* and *ku80* KO strains. We first cultivated the *ku80* KO and Δ*PlwetA* strains at different temperatures (22°C, 26°C, 30°C, and 34°C). The conidial germination rate of the Δ*PlwetA* strain was significantly lower at 26°C and 30°C than that of the *ku80* KO strain (Fig. 5B and C). When cultured at 22°C and 34°C, the germination rates of the conidia of both strains were very low (Fig. 5A and D). Remarkably, the conidium germinations of the two strains were similar, initiated by isotropic swelling of conidia and followed by polarization and emergence of a hyphal germ tube in the mutant strain. However, in the *ku80* KO strain, conidia swelled bigger than in the mutant (Fig. S9).

**FIG 6:**
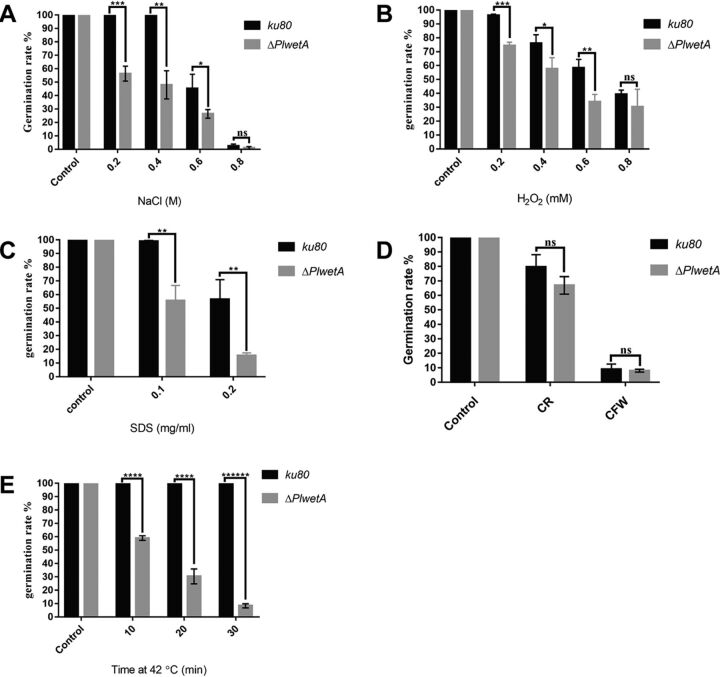
The deletion of *PlwetA* resulted in higher sensitivity to various stresses in *P. lavendulum*. (A) High osmotic stress (0.2 to 0.8 M NaCl); (B) oxidative stress (0.2 to 0.8 mM H_2_O_2_); (C) 0.1 and 0.2 mg/ml of sodium dodecyl sulfate; (D) 0.2 mg/ml of Congo red and 10 μg/ml of calcofluor white; (E) heat shock at 42°C for 10, 20, or 30 min followed by growth at 28°C. *, *P* < 0.05; **, *P* < 0.01; ***, *P* < 0.001; ****, *P* < 0.0001; ******, *P* < 0.000001; ns, not significant.

We then tested the effects of *PlwetA* on spore tolerance to various stresses and found that ΔPl*wetA* mutant conidia exhibited drastically reduced tolerance to osmotic and oxidative stresses and were susceptible to the cell wall-interfering agents sodium dodecyl sulfate (SDS), Congo red (CR), calcofluor white (CFW), and heat shock (Fig. 6). The mutant was especially sensitive to heat (heated at 42°C for 10, 20, or 30 min) (Fig. 6E). Taken together, these results suggest that *PlwetA* plays an essential role in the proper maturation and stress tolerance of conidia in *P. lavendulum*.

### Deletion of *PlwetA* caused a defect in trehalose production.

**FIG 7:**
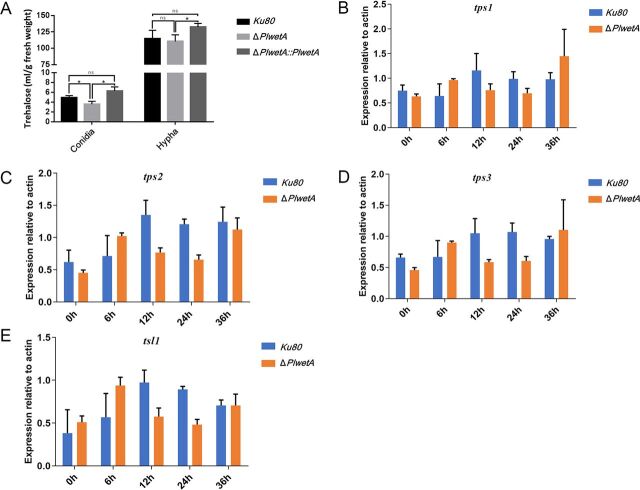
The deletion of *PlwetA* led to reduced trehalose production in conidia in *P. lavendulum*. (A) The amount of trehalose in conidia and hyphae of the *ku80* KO strain, the *PlWetA* mutant, and the complemented strain; (B to E) RT-qPCR analysis of trehalose synthetic genes in the *ku80* KO and Δ*PlwetA* strains. *, *P* < 0.05; ns, not significant.

Trehalose is known to be associated with the viability of conidia and stress resistance (10, 11). We measured trehalose production in spores and hyphae of the *ku80* KO, Δ*PlwetA*, and Δ*PlwetA*::*PlwetA* strains. We found that the Δ*PlwetA*, Δ*PlwetA*::*PlwetA*, and *ku80* KO strains produced similar amounts of trehalose in hyphae (Fig. 7A). Yet in conidia, the Δ*PlwetA* strain contained a lower trehalose content than that in the *ku80* KO strain and its complemented Δ*PlwetA*::*PlwetA* strain, consistent with a relationship between spore defect and trehalose content.

Loss of canonical trehalose biosynthesis genes in *Aspergillus* significantly alters cell wall structure and integrity (12). To identify putative regulatory subunits of the trehalose complex in *P. lavendulum*, the orthologous proteins of Saccharomyces cerevisiae, Tps1, Tps2, Tps3, and Tsl1, were identified in *P. lavendulum* using BLASTp searches. The amino acid sequences of PlTps1, PlTps2, and PlTps3 showed 65%, 41%, and 39% identities to their S. cerevisiae orthologs, respectively. Protein domain analyses revealed that Tps1 and Tps2 shared domains similar to those of trehalose-6-phosphate synthase and trehalose-6-phosphatase, respectively. To explore the molecular mechanisms of the deficiency of conidia underlying PlWetA regulation, expression levels of *Pltps1*, *Pltps2*, *Pltps3*, and *Pltsl1* during conidiation were examined by RT-qPCR (Fig. 7B to E). In both the *ku80* KO and Δ*PlwetA* strains, the expression levels of all four genes were elevated after 6 h of conidiation induction and declined after 12 h (*Pltps1*, *Pltps2*, and *Pltsl1*) or 24 h (*Pltps3*). The expression levels of these genes in the Δ*PlwetA* strain were lower than that in the *ku80* KO strain at the 12- and 24-h time points. This expression pattern suggested that trehalose production was regulated by PlWetA.

### PlBrlA and PlAbaA, but not PlWetA, are dispensable for the secondary metabolites leucinostatins A and B in *P. lavendulum*.

**FIG 8:**
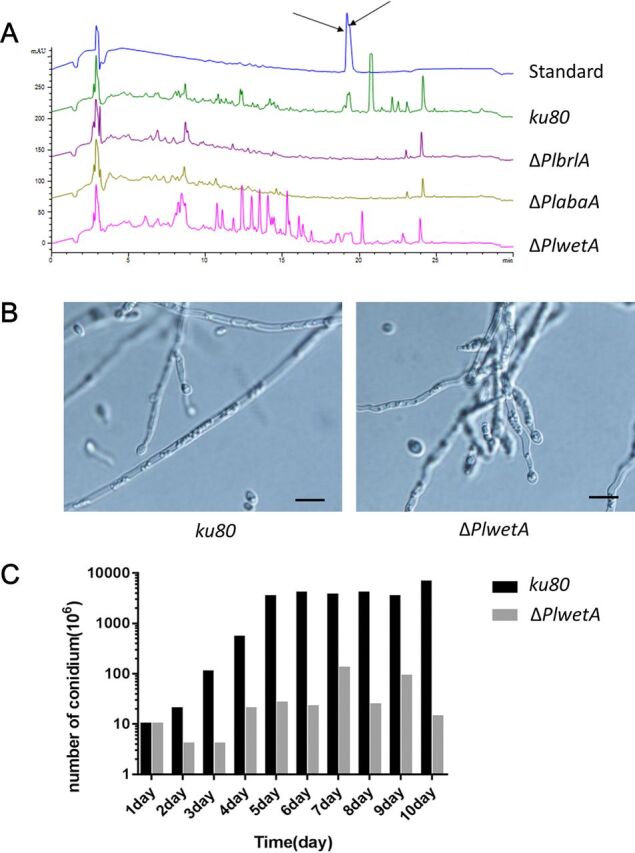
(A) HPLC detection of leucinostatins A and B in the *ku80* KO, Δ*PlbrlA*, Δ*PlabaA*, and Δ*PlwetA* strains. Black arrows indicate the leucinostatin A and B peak. (B) Observation of conidium production in liquid media in the *ku80* KO and Δ*PlwetA* strains. Bar = 10 μm. (C) Comparison of conidium numbers between the *ku80* KO and Δ*PlwetA* strains cultivated in liquid medium for 10 days.

The fungal conidiation process is accompanied by many changes, including that of secondary-metabolite production. A previous report indicated that leucinostatins A and B produced in *P. lilacinum* could cause strong mortality and inhibited nematode reproduction (13). The leucinostatin biosynthesis gene cluster has been identified. In this study, we investigated whether *PlBrlA*, *PlAbaA*, or *PlwetA* was involved in the production of leucinostatins. We examined leucinostatin A and B production in the *ku80* KO, Δ*PlbrlA*, Δ*PlabaA*, and Δ*PlwetA* strains using mycelia from a 10-day-old shaker culture by high-performance liquid chromatography (HPLC) (Fig. 7A). The results showed that both the *ku80* KO and the Δ*PlwetA* strains accumulated leucinostatins, while the Δ*PlbrlA* and Δ*PlabaA* strains did not produce detectable leucinostatins, in liquid culture. Interestingly, many other unknown secondary metabolites were also decreased dramatically in the last two strains. Our data showed that the expressions of both *PlbrlA and PlabaA* are necessary for leucinostatin biosynthesis. Besides the RT-qPCR performed above, we examined whether conidia were produced in the liquid cultures to confirm the expression of conidiation-related genes. The spores in the liquid medium were observed and counted after 48 h of inoculation, followed by every 24 h, until the 10th day. As shown in Fig. 8B and C, the *ku80* KO and Δ*PlwetA* strains both produced conidia in submerged cultures, with the *ku80* KO strain producing more conidia than the Δ*PlwetA* strain.

### The conidiation-related genes are not directly involved in fungal pathogenesis against root-knot nematode eggs.

**FIG 9:**
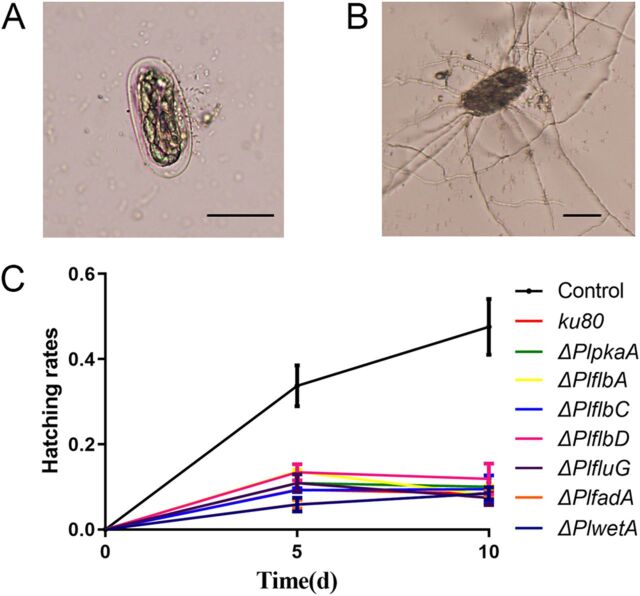
Bioassay against root-knot nematode eggs. (A) An intact nematode egg; (B) a nematode egg infected by *P. lavendulum*; (C) the hatching rates after fungal infection for various times. Bar = 50 μm.

To further evaluate the potential effects of these genes in fungal pathogenesis against nematodes, bioassays against the eggs of the root-knot nematode Meloidogyne incognita were carried out in the *ku80* KO and other derived mutant strains (Fig. 9). About 30% and 50% eggs had hatched within 5 and 10 days in the control set (without fungal infection), while in the infection sets, less than 10% were hatched (Fig. 9C). However, no significant differences were found between the *ku80* KO strain and other mutants. As we used the same amounts of spores in the assays, the result revealed that although these conidium regulation genes were not directly involved in fungal pathogenesis against root-knot nematode eggs, they do play a role in fungal pathogenesis by regulating the quality and quantity of conidium production.

## DISCUSSION

In this study, we identified the regulatory genes in the conidiation of the nematophagous fungus *P. lavendulum* and investigated their biological effects through systematic gene deletion analyses. Our focus was on dissecting the developmental process of conidiogenesis. Several key development regulators were identified which could help enhance the biocontrol efficacy of this fungus against nematodes.

The orthologs of PlBrlA, PlAbaA, and PlWetA make up the central regulation module of conidiation in A. nidulans. They control the initiation and the middle and late stages of conidiogenesis. From our previous study, knocking out the *PlbrlA* gene in *P. lavendulum* totally blocked the formation of conidiophores (8). In the Δ*PlbrlA* mutant, the hyphae were extremely elongated, and the colony was loose and white. Disruption of the *PlabaA* gene also resulted in aborted conidiation. The ultrastructural study showed that *PlabaA* mutants produced immature phialides with a long tube-like structure but had no differentiated conidia (Fig. 2E). This phenotype is a little bit different from that in A. nidulans, which formed abacus-like structures (14, 15). Meanwhile, although deletion of *PlwetA* did not inhibit the initiation of conidiation, the conidia of the *PlwetA* mutant were immature. The cell walls of conidia were aberrant, with vacuoles and shallow pigmentation, and the germination of these conidia was delayed. The number of conidia was significantly lower than in the *ku80* KO strain and the complemented strain. Collectively, the results suggest that like in other fungal species, the three regulators PlBrlA, PlAbaA, and PlWetA play a central role in conidiation regulation in *P*. *lavendulum*.

Genetic studies showed that several upstream developmental activators (the fluffy genes *fluG*, *flbA*, *flbB*, *flbC*, *flbD*, and *flbE*) are required for activation of the central regulatory pathway and play important roles in conidiation initiation in A. nidulans (16, 17). In parallel to the conidiation signaling cascade, two main G-protein-mediated signaling pathways, FadA (Gα) and SfaD (Gβ)::GpgA (Gγ) and GanB (Gα) and SfaD (Gβ)::GpgA (Gγ), can negatively influence fungal development and *brlA* expression via modulating cAMP/protein kinase A (PKA) signaling (7, 18). The FadA and SfaD::GpgA pathway activates vegetative growth and represses conidiation (18–20). Surprisingly, besides the *PlflbA* gene, knocking out the fluffy genes *PlflbC* and *PlflbD* did not cause decreases in conidia. This result was different from that for the homologous genes in A. nidulans. The specific functions of *PlflbA*, *PlflbC*, *PlflbD*, and *PlfluG* require further study.

The conidium maturation regulated by PlWetA also affected conidium germination under both normal and stressful culture conditions. Loss-of-function mutations in the *wetA* locus in A. nidulans and Aspergillus fumigatus led to defects in the crenulation of the C1 wall layer and in condensation of the C2 wall layer in conidia (11, 21). Similarly, crenulation of the C1 wall layer was observed in the Δ*PlwetA* mutant and the C2 layer in the Δ*PlwetA* mutant turned quite loose and thick. Meanwhile, large vacuoles were found inside the conidia, indicating an additional role besides the conidial wall completion in *P*. *lavendulum*. In A. fumigatus and Fusarium graminearum, the conidia of the *wetA* ortholog deletion mutant had a survival rate of less than 10% at 20 days after incubation (11, 22). However, in spite of delayed germination for Δ*PlwetA* conidia, about 70% conidia could finally germinate after 13 h of incubation. A stress tolerance test indicated that conidia of the Δ*PlwetA* strain were more sensitive to osmotic stress, SDS, CR, CFW, heat shock, and oxidative stress. These results were likely due to deficient conidia produced by the *PlwetA* deletion mutant.

Trehalose is now recognized to serve highly diverse functions for prokaryotic and eukaryotic cells, ranging from cellular protection to symbiotic and pathogenic host interactions (23). The lack of trehalose in fungal spores and hyphae often leads to rapid loss of viability and increased sensitivity to a variety of physiological stresses (10, 24, 25). In aspergilli, such as A. nidulans and A. fumigatus, trehalose is known to be required for spore viability and stress resistance, and *wetA* is required for trehalose production in A. fumigatus (10, 11, 26). However, there was no significant difference in trehalose content in hyphae between the *ku80* KO and Δ*PlwetA* strains. In contrast, the spores of the Δ*PlwetA* mutant contained much less trehalose than the *ku80* KO strain. The mRNA levels of some trehalose biosynthesis genes were analyzed by RT-qPCR. Our results suggest that PlWetA expression was likely involved in regulating the biosynthesis of trehalose. Together, these results revealed that though the WetA orthologs have highly conserved functions, additional functions may have evolved for this gene in *P*. *lavendulum*.

The central regulatory pathway of conidiation could also influence secondary-metabolite production. The production of secondary metabolites in some nematophagous fungi has been linked to their nematicidal activities. For example, culture filtrates of *P. lilacinum*, in which leucinostatin was produced, had nematicidal activity and inhibited nematode reproduction (13). The gene cluster of leucinostatin production in *P. lilacinum* had been identified and characterized (27). We also found that a similar gene cluster existed in the *P*. *lavendulum* genome. Thus, we speculated whether leucinostatin was produced in *P*. *lavendulum* and correlated leucinostatin production to conidiation. Using HPLC analysis, we found that leucinostatins A and B were produced in the liquid culture of *P*. *lavendulum*. The Δ*PlwetA* strain seemed to produce more leucinostatins as well as other secondary metabolites, while the production of leucinostatins was inhibited in the Δ*PlbrlA* and Δ*PlabaA* strains. These results implied that *PlbrlA* and *PlabaA* were required for the expression of the gene cluster involved in leucinostatin production. The expression of *PlbrlA*, *PlabaA*, and *PlwetA* in liquid-cultured hyphae was indicated by conidium production, although the amount of conidia was very low compared with that of organisms cultured on solid agar media (Fig. 8). The bioassays against root-knot nematode eggs showed no direct roles of these genes, as well as leucinostatins A and B (maybe due to low production under such conditions), in fungal pathogenesis. However, the decreased spore amounts and the decreased abilities of the mutants to survive environmental stresses indicate their indirect roles in their biocontrol efficiencies against nematodes.

**FIG 10:**
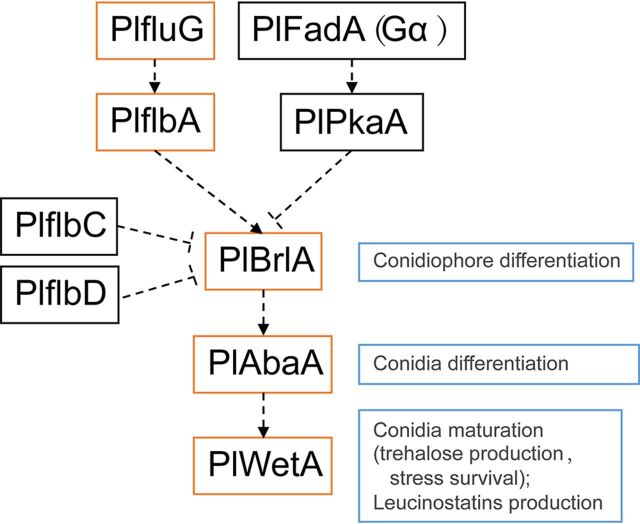
A putative framework for gene regulation in *P. lavendulum* conidiation. Orange rectangles indicate positive regulators in conidiation, and black rectangles indicate negative ones. The arrangement and relationship between regulators were based on their homologs in A. nidulans.

In this study, we functionally characterized several conidiation regulation genes in *P*. *lavendulum* and composed a basic framework of *P*. *lavendulum* conidiogenesis (Fig. 10). These results expand our knowledge of conidiogenesis and conidium maturation in filamentous fungi. However, a great deal of information remains to be obtained, such as the molecular processes that regulate *PlbrlA*, *PlabaA*, and *PlwetA* expression and the downstream genes that are directly regulated by these three genes. Further studies are needed to reveal the genetic network underlying conidiogenesis in *P*. *lavendulum* and the metabolic processes involved in its development.

## MATERIALS AND METHODS

### Strains, vector, and bacterial strains.

*Purpureocillium lavendulum* wild-type strain YMF1.00683 was isolated from soil in Yunnan Province, China. The *ku80* gene, involved in the nonhomologous end joining (NHEJ) pathway, was knocked out in this strain to create a deletion mutant which showed a high efficiency in homologous recombination (8). This deletion mutant was the main strain used for subsequent molecular analyses in this study. Both the *ku80* KO strain and its derived knockout mutant strains were maintained in minimal solid or liquid medium (MM) with 2% glucose and incubated at 28°C (8). Escherichia coli DH5α was used for vector construction. Agrobacterium tumefaciens AGL-1 was used for fungal transformation (28). Strains used in this study are described in Table 1.

**TABLE 1:** Strains used in this study

Strain description	Knockout gene(s)	Reference or source
*ku80*	*ku80*	8
Δ*PlbrlA*	*PlbrlA*, *ku80*	8
Δ*PlabaA*	*PlabaA*, *ku80*	This study
Δ*PlwetA*	*PlwetA*, *ku80*	This study
Δ*PlfadA*	*PlfadA*, *ku80*	This study
Δ*PlpkaA*	*PlpkaA*, *ku80*	This study
Δ*PlflbA*	*PlflbA*, *ku80*	This study
Δ*PlflbC*	*PlflbC*, *ku80*	This study
Δ*PlflbD*	*PlflbD*, *ku80*	This study
Δ*PlfluG*	*PlfluG*, *ku80*	This study

### Construction of mutant strains.

To search for putative sporulation genes in the genome of *P. lavendulum* for our investigation, we first used the sequences of the core regulatory genes and the fluffy genes involved in the sporulation in A. nidulans as queries and compared them to the genome sequence of *P. lavendulum* by BLASTp search (29).

Gene knockouts were performed by the homologous recombination strategy. Knockout plasmids were generated by the Gateway-based method described previously (8). Briefly, the 5′ and 3′ homologous arms of the candidate sporulation genes were PCR amplified with a specific *attB* recombination site added at each 5′ end. Then the two arms and two plasmids, pPK2-OSCAR-GFP and pA-Sur-OSCAR, were assembled by Gateway cloning with the BP clonase II enzyme mix (Invitrogen, Thermo Fisher Scientific). The assembled knockout plasmids were transformed into the *P. lavendulum ku80* strain by A. tumefaciens-mediated transformation. Colonies grown on selective medium containing 50 μg/ml of chlorimuron were then screened by gene-specific PCR and confirmed by Southern hybridization.

To obtain a complemented strain, a genomic DNA fragment of the target wild-type gene including the promoter region, ORF, and termination region was PCR amplified and inserted into plasmid pPK2-bml-GFP (cut by SbfI/XbaI). The reconstructed plasmid was then transformed into the corresponding mutant strain by A. tumefaciens-mediated transformation. The transformants were selected on MM agar containing 10 μg/ml of benomyl. The colonies were screened by PCR and confirmed by Southern blotting. Primers used in gene knockouts, complementary plasmid constructions, PCR screening, and Southern blotting are listed in Table S1.

10.1128/mSphere.00932-20.10 TABLE S1 PCR primers used in this study. Download Table S1, DOCX file, 0.03 MB. Copyright © 2020 Chen et al. 2020 Chen et al. This content is distributed under the terms of the Creative Commons Attribution 4.0 International license.

### Quantitative real-time PCR analysis.

Total RNA from the *P. lavendulum* was isolated as described for a previous study (30). cDNA was generated by reverse transcription of the total RNA using SuperScript II (Invitrogen). Real-time quantitative PCR (RT-qPCR) analysis was performed using an ABI Prism 7000 system (Applied Biosystems Inc., Norwalk, CT) using SYBR green (TaKaRa, Dalian, China) as described previously (31). The actin gene was used as the reference gene. The primers used for RT-qPCR analysis are listed in Table S1.

### Conidium production and morphology comparison.

Conidium production was quantified by counting the number of conidia produced after culturing 5 μl of a conidial suspension (1 × 10^7^ conidia/ml) of each strain on solid MM agar at 28°C for 8 days. After incubation, three 0.8-cm-diameter cores were harvested from the center of each plate and homogenized in 1 ml of 0.05% Tween. Counting was performed with a hemocytometer. For colony phenotype, a point inoculation of 2.5 μl of 1 × 10^4^ spores/ml was performed on MM agar and incubated for 10 days at 28°C, followed by colony phenotype observations. In addition, we used another method of spore counting for individual conidiophores. Here, each strain was inoculated onto a thin layer of solid MM agar sandwiched between a sterilized slide and a coverslip. The slides were then placed on top of glass rods in a moist plate containing 20% glycerol in water (moist chamber culture). After 8 days of incubation, the number of spores on each conidiophore was counted under a light microscope.

### Stress tolerance tests and trehalose assay.

To evaluate the effects of gene knockouts on conidium germination under various stress conditions, germination rates of conidia from the *ku80* deletion mutant and other mutants were inoculated onto solid MM supplemented with various concentrations of NaCl (0.2 to 0.8 M), H_2_O_2_ (0.2 to 0.8 mM), sodium dodecyl sulfate (SDS; 0.1 to 0.2 mg/ml), Congo red (CR; 0.2 mg/ml), or calcofluor white (CFW; 10 μg/ml). To evaluate thermal tolerance, conidia were first exposed to 42°C for various times and then transferred onto MM and incubated at 28°C to obtain germinated spore counts. Moreover, conidia from all strains were inoculated onto MM and cultured at different temperatures, with germination rates determined at 8, 9, 10, 11, 12, and 13 h postinoculation. All experiments were conducted twice, with three replicates for each strain in each experiment.

The trehalose assay was performed as described previously (22), with slight modifications. Briefly, freshly harvested conidia (1 × 10^8^ conidia from a 10-day-old culture) were inoculated onto liquid MM and incubated at 28°C for 2 days on a rotary shaker (140 rpm). Approximately 8 mg of hyphae/conidia were washed three times with 0.05% Tween 20, resuspended in 1 ml of extraction solution, and incubated at 100°C for 3 h. After centrifugation for 10 min at 11,000 × *g*, 250 μl of supernatant was mixed with 1 ml of reaction mixture and samples were incubated at 95°C for 10 min. The samples were then transferred to room temperature to measure trehalose content by using a trehalose assay kit (Solarbio, Beijing, China) according to the manufacturer’s instructions. The experiments were performed independently three times.

### Electron microscopy.

To determine the effects of gene deletions on hyphal structure, the original strain and the derived deletion mutants were grown in MM for 15 days at 28°C. The conidiophore structures were investigated by scanning electron microscopy (SEM) as described previously (32). The cell walls of the *ku80* KO and the ΔPl*wetA* strains were examined by transmission electron microscopy (TEM) as described previously (33).

### Measurement of leucinostatins.

The *P. lavendulum ku80* KO strain and three deletion mutants, the Δ*PlwetA*, Δ*PlabaA*, and Δ*PlbrlA* mutants, were cultivated in 200 ml of PDB medium (potato dextrose broth, potato starch [from infusion] 4.0 g/liter, dextrose 20.0 g/liter) at 28°C on a shaker at 140 rpm for 10 days. The culture medium (200 ml) was extracted with the same volume of ethyl acetate two times (each time for 20 min) ultrasonically with vortexing for 20 min. The organic phases were combined and dried with methyl alcohol and acetone three times. The samples were redissolved in 0.5 ml of methanol and measured on an Agilent HPLC instrument. For HPLC measurements, 10 μl of the standard leucinostatin A-B mixture or extracted sample was injected. A ZORBA×ODS (4.6 by 205 mm; 5 μl) column was used at a flow rate of 1 ml/min with the following gradient: fresh extracts of mutant strains were detected for 30 min using a linear gradient of 20% to 100% methyl alcohol (0 to 20 min), 100% methyl alcohol (20 to 25 min), and 20% methyl alcohol (25 to 30 min) (27).

### Biocontrol activity assay against the root-knot nematode Meloidogyne incognita.

To identify the potential role of sporulation-related genes in infection of nematodes in *P. lavendulum*, we assayed the infectivity of mutant strains against the root-knot nematode, Meloidogyne incognita. Briefly, 10^7^ conidia of each strain was added to 200 newly isolated eggs of Meloidogyne incognita suspended in 2 ml of distilled water in a 5-cm petri dish and incubated at 28°C for 10 days. The hatched nematodes were counted under a light microscope. The assays were performed three times.

### GenBank accession numbers.

The sequences for the *P. lavendulum* genes newly identified in this study have been deposited into GenBank under the following accession numbers: *PlfadA*, MK603194; *PlpkaA*, MK603195; *PlflbA*, MK603196; *PlflbC*, MK603197; *PlflbD*, MK603198; and *PlfluG*, MK603199.
