# Supplementary figures and images for: Functional Characterization of Core Regulatory Genes Involved in Sporulation of the Nematophagous Fungus Purpureocillium lavendulum
Source: mSphere. 2020 Oct 28;5(5):e00932-20. doi: 10.1128/mSphere.00932-20 (PMC8534313; doi:10.1128/mSphere.00932-20)

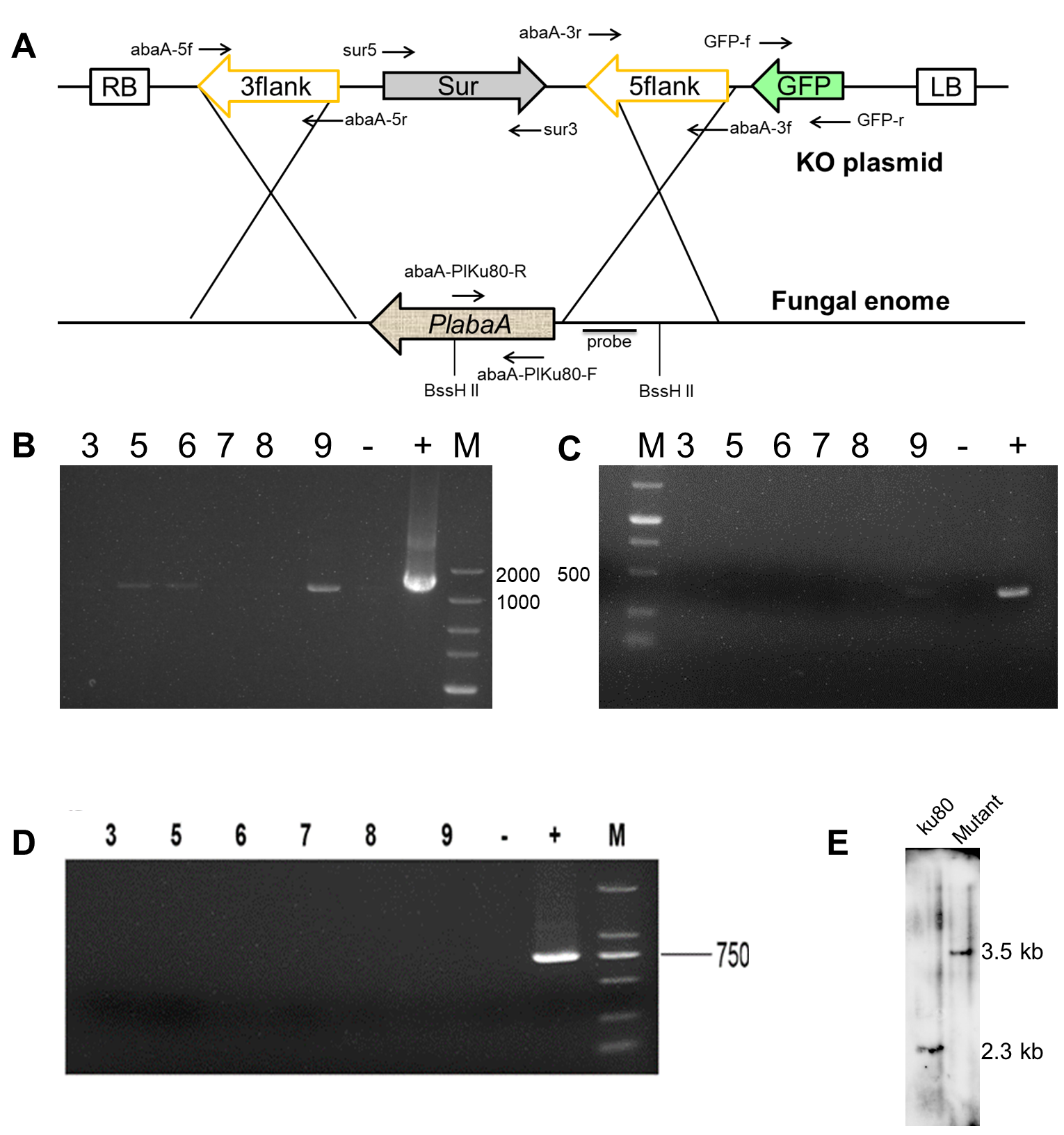

Supplement: FIG S1 [file msphere.00932-20-sf001.tif]

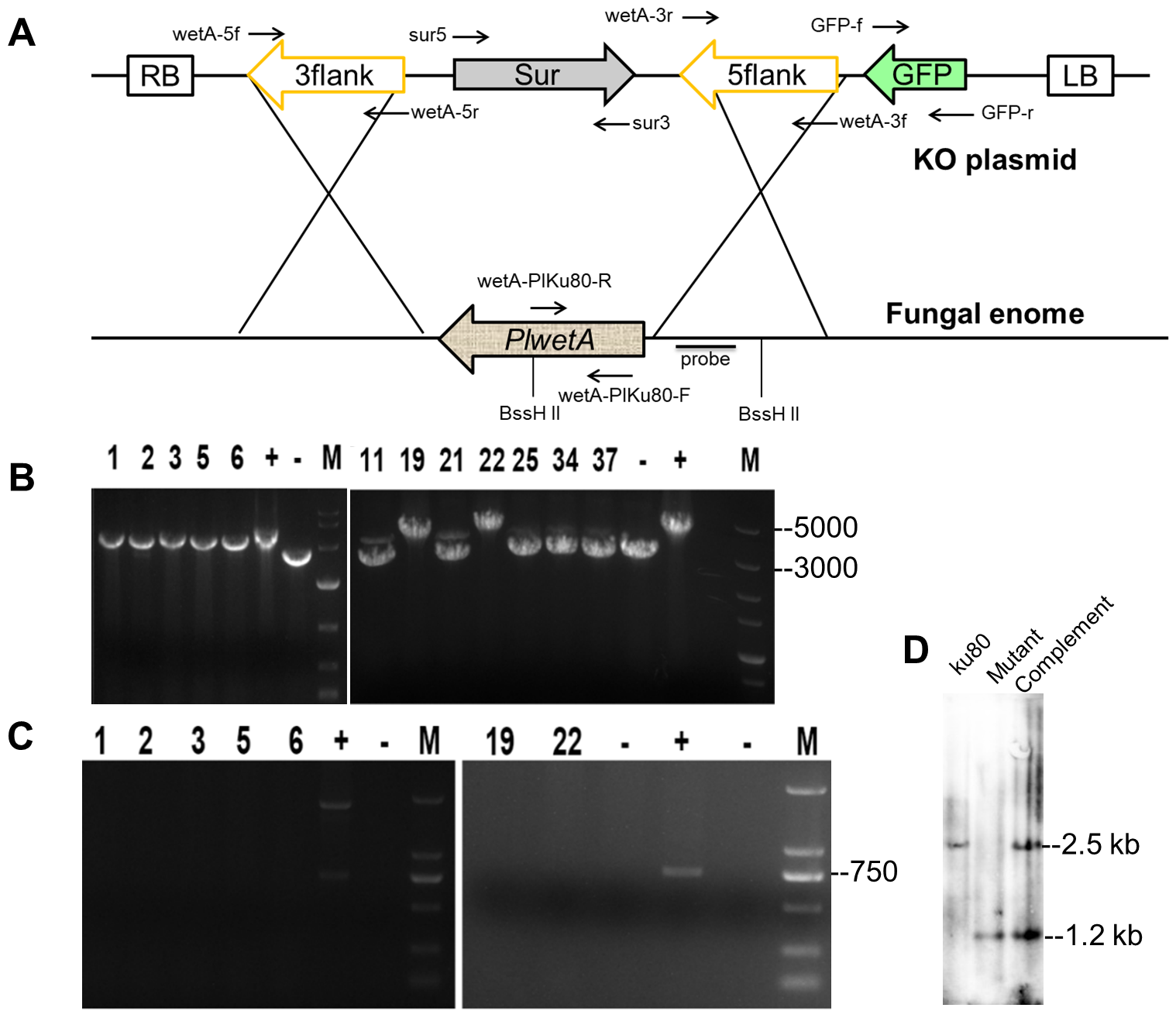

Supplement: FIG S2 [file msphere.00932-20-sf002.tif]

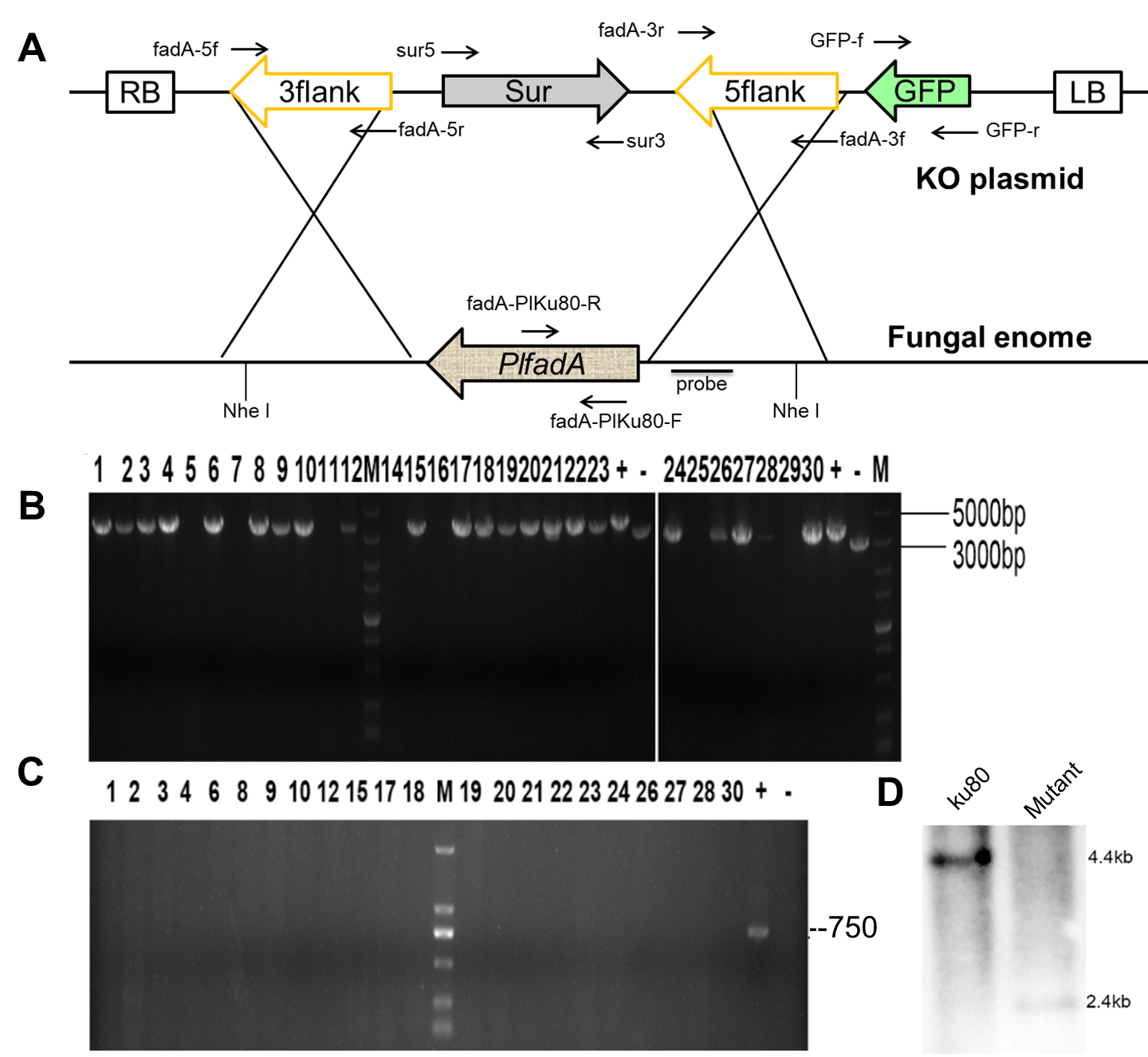

Supplement: FIG S3 [file msphere.00932-20-sf003.tif]

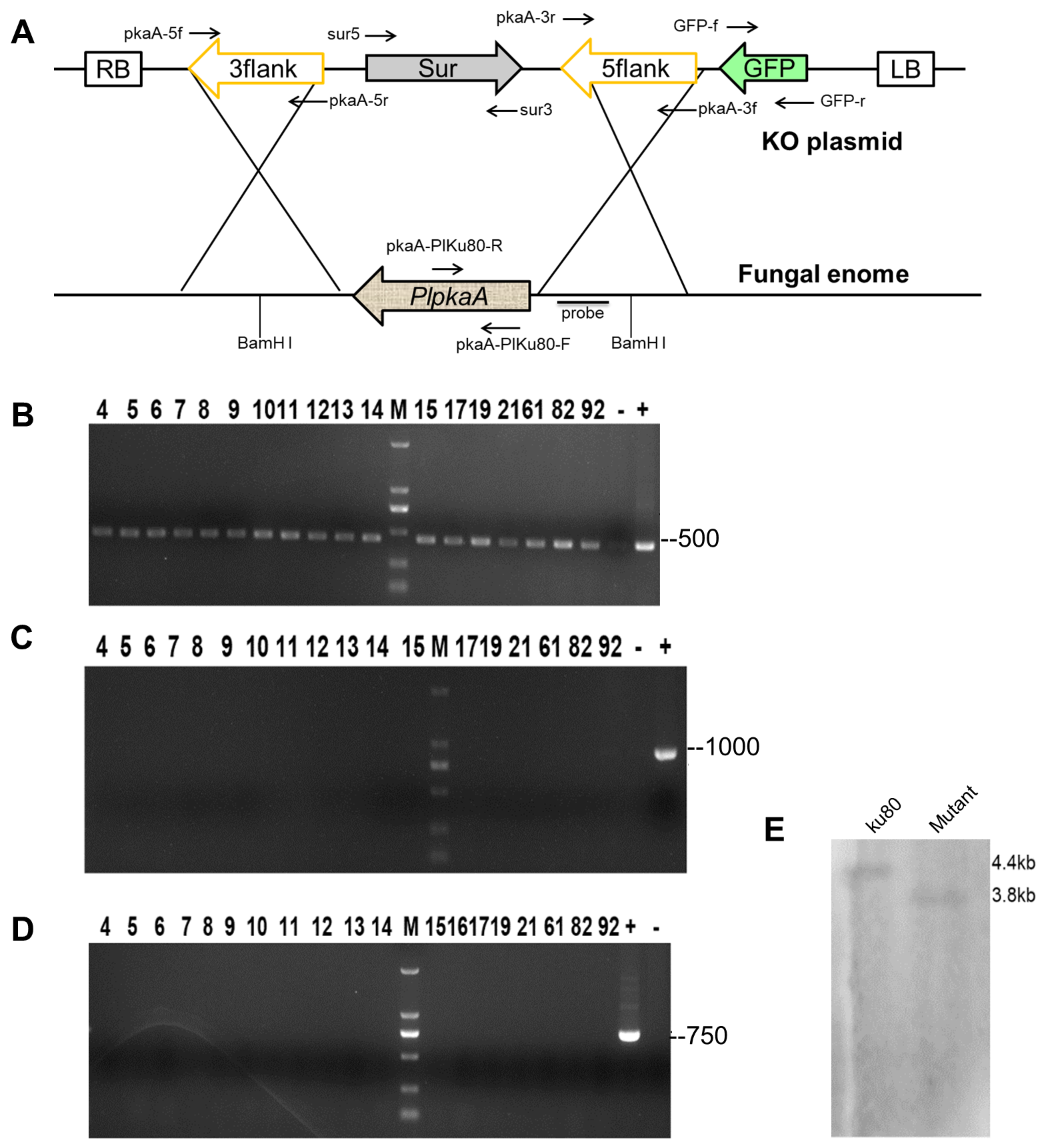

Supplement: FIG S4 [file msphere.00932-20-sf004.tif]

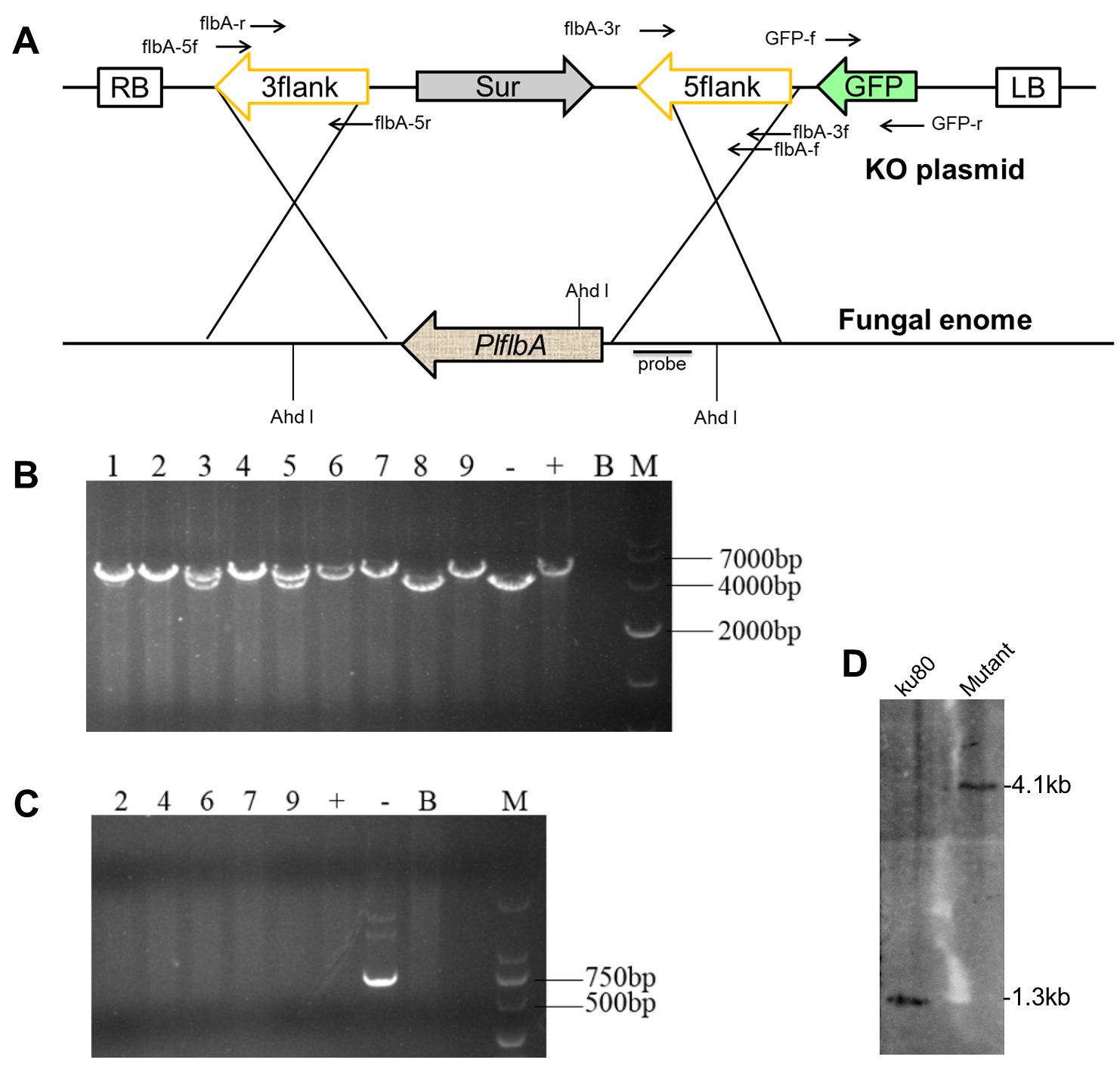

Supplement: FIG S5 [file msphere.00932-20-sf005.tif]

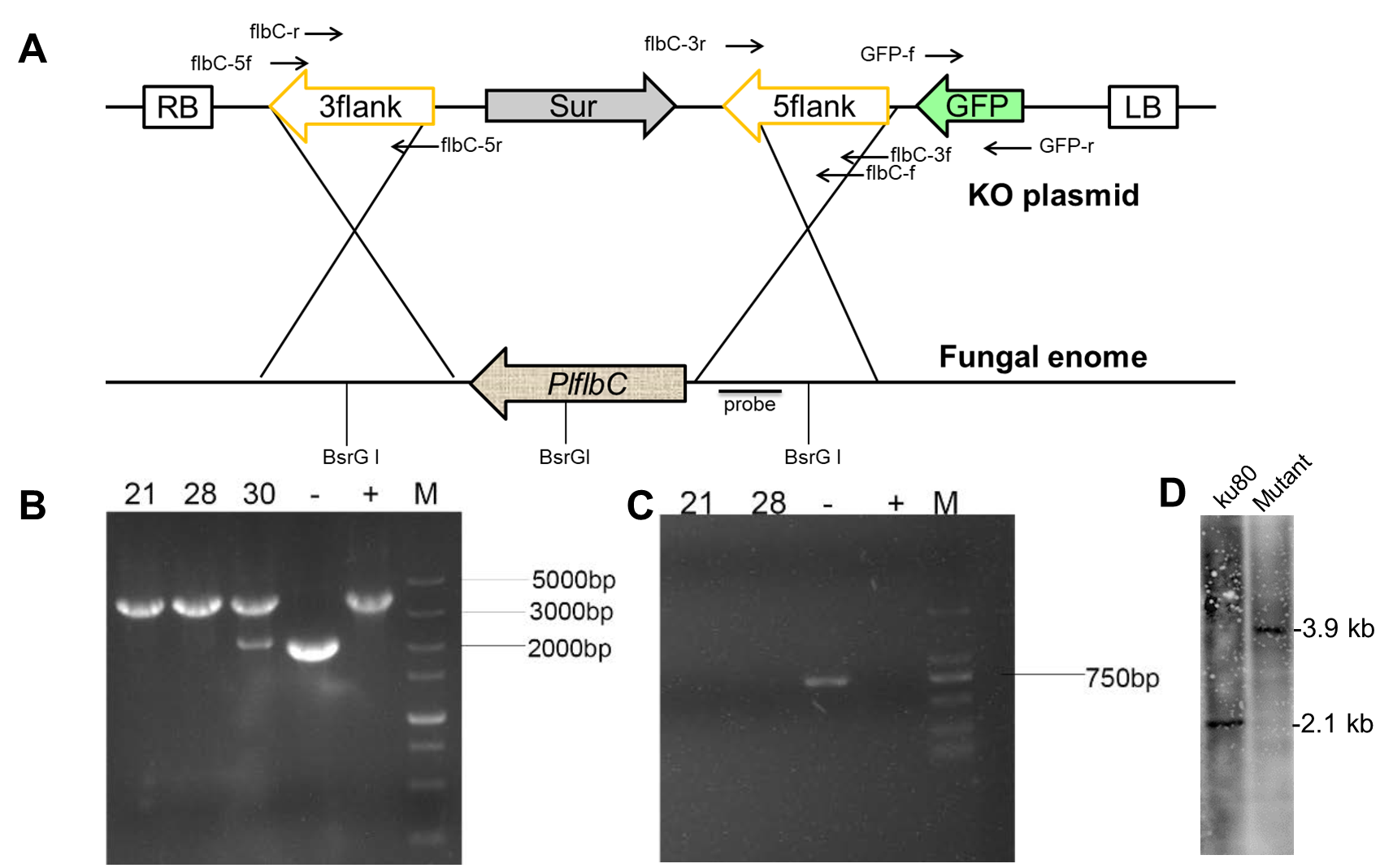

Supplement: FIG S6 [file msphere.00932-20-sf006.tif]

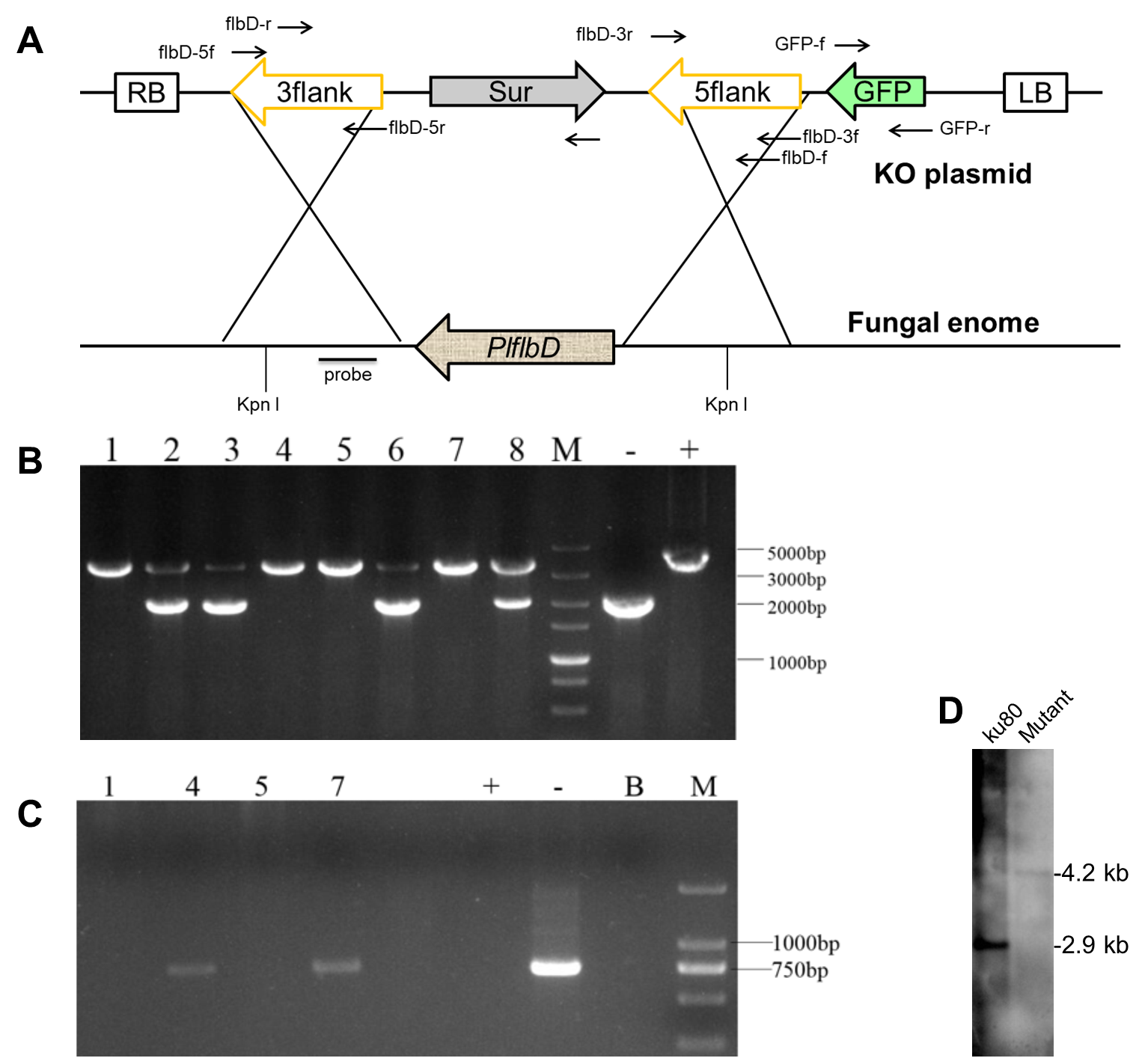

Supplement: FIG S7 [file msphere.00932-20-sf007.tif]

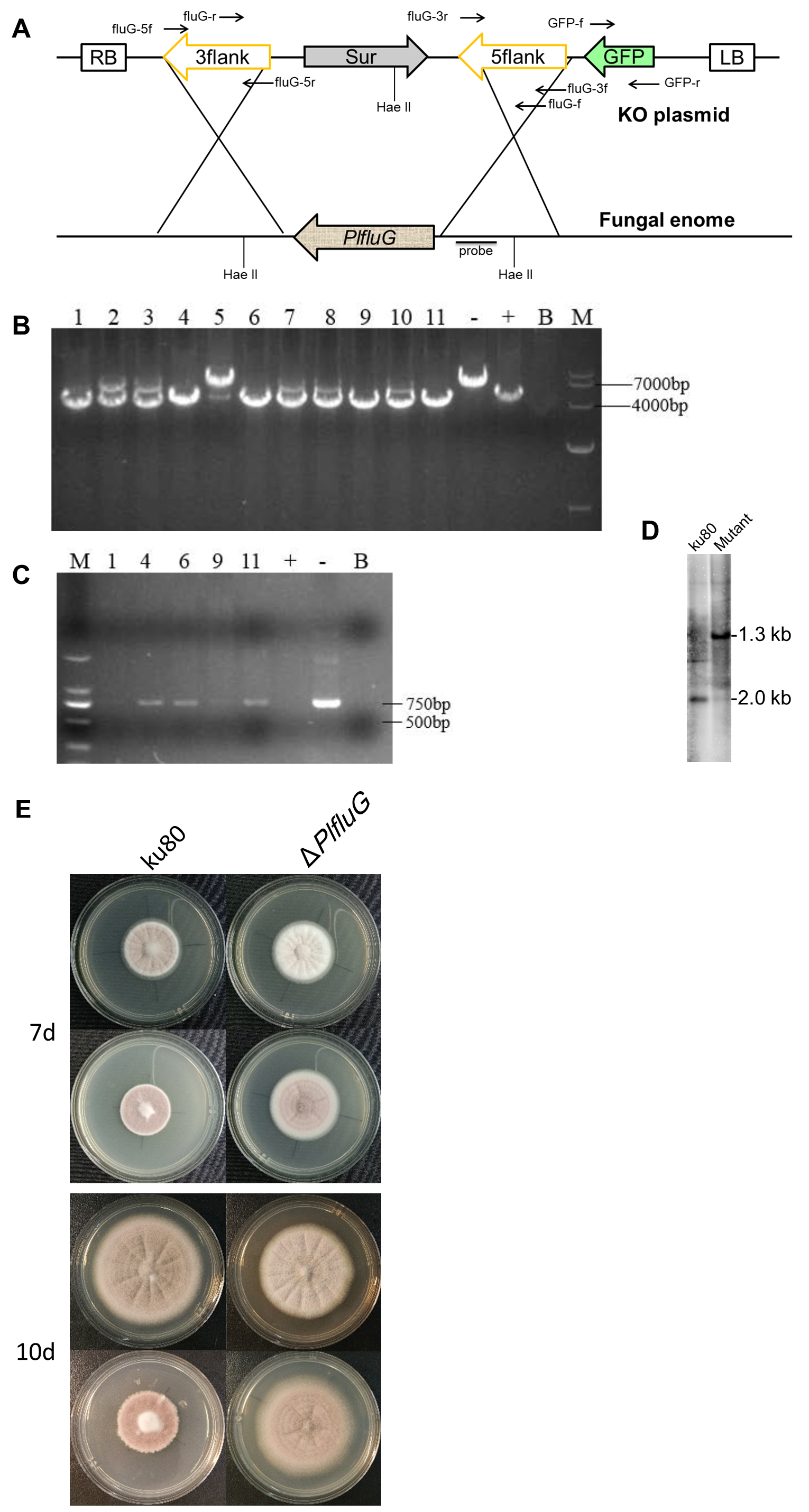

Supplement: FIG S8 [file msphere.00932-20-sf008.tif]

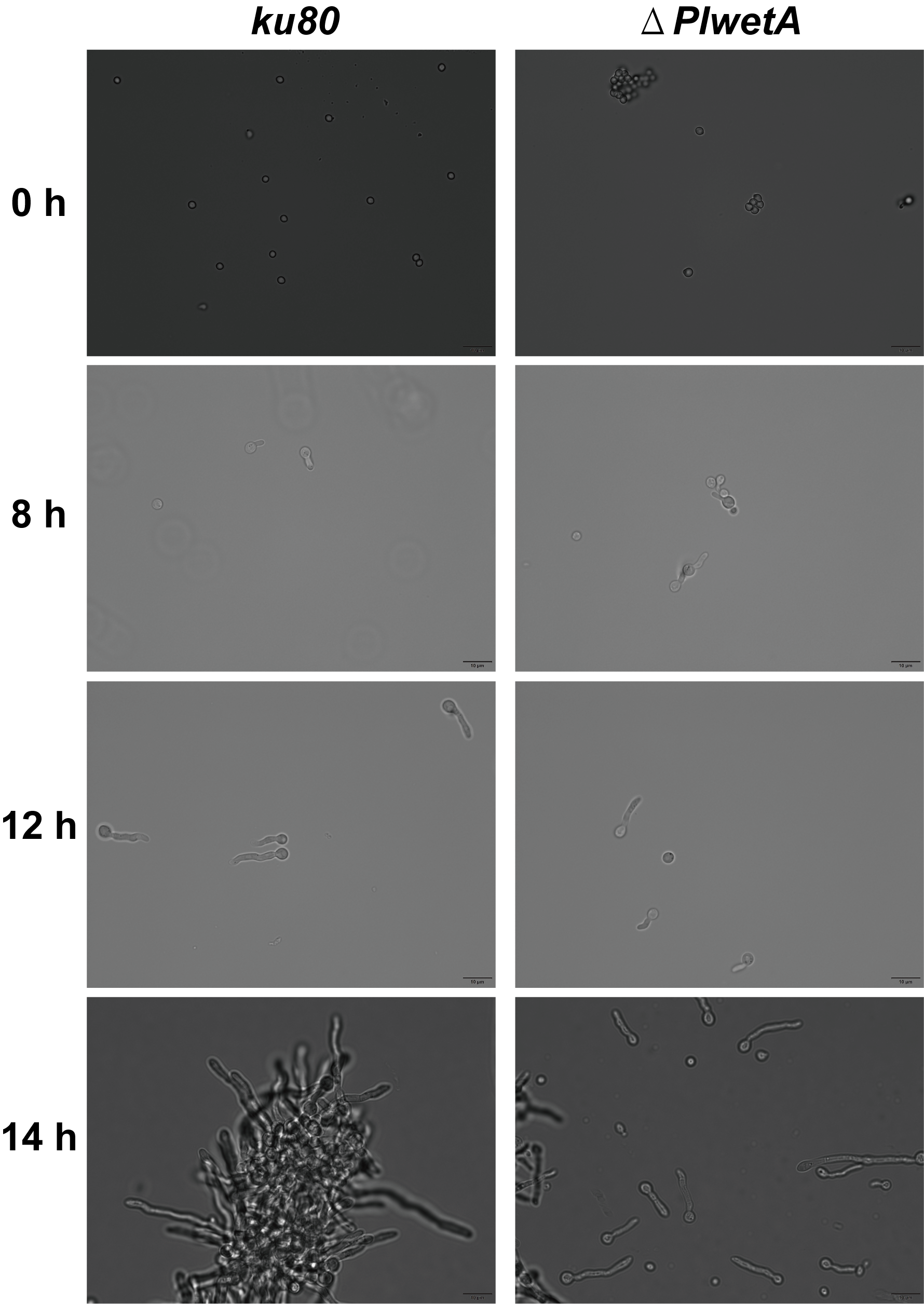

Supplement: FIG S9 [file msphere.00932-20-sf009.tif]
