# Supplementary material for: Functional Characterization of Core Regulatory Genes Involved in Sporulation of the Nematophagous Fungus Purpureocillium lavendulum
Source: mSphere. 2020 Oct 28;5(5):e00932-20. doi: 10.1128/mSphere.00932-20 (PMC8534313; doi:10.1128/mSphere.00932-20)
Supplement: TABLE S1 [file msphere.00932-20-st001.docx]

**Supplemental Table S1. PCR primers used in this study**

| **Primer** | **Sequence (5ʹ – 3ʹ)** | **Description** |
| --- | --- | --- |
| ApaI5f-PlKu80 | cacgctagagctgcagggcccGTCACCGTGTTACCTTCCTCTG | For cloning the full gene of *PlWetA* for gene complementation. |
| AflII3r-PlKu80 | aaaacgacggccagtcttaagGTACCACCAAGCTCTGCTGTCT |  |
| bml-5f | GGAGCCTGAATGTTGAGTG | For amplification of bml gene to identify positive clones of *PlWetA* complementation. |
| bml-3r | GAACCGAGCCAGAGTGC |  |
| GFP-F | TTCACCTTGATGCCGTTCT | For amplification of GFP gene fragment to identify ectopic insertion. |
| GFP-R | CCACAAGTTCAGCGTGTCC |  |
| sur-5F | TGACTCCACGATTCACAGC | For amplification of sur gene fragment to identify positive transformants. |
| sur-3R | CCATTGGGTCACCATTCC |  |
| pkaA-5f | ggggacagctttcttgtacaaagtggaaACCTGTTAGTCGGTACGTCAAAA | For amplification of the *PlPkaA* gene 5’ flank |
| pkaA-5r | ggggactgcttttttgtacaaacttgtCACTGCTGTATGTTGATGATGGT |  |
| pkaA-3f | ggggacaactttgtatagaaaagttgttAGAAATGGCTCCTCTCGATTTAC | For amplification of the *PlPkaA* gene 3’ flank |
| pkaA-3r | ggggacaactttgtataataaagttgtATGTGTCCAATTGTAGTCCTTGG |  |
| fadA-5f | ggggacagctttcttgtacaaagtggaaCCTAGACAAGGTTGTTCAGATGG | For amplification of the *PlFadA* gene 5’ flank |
| fadA-5r | ggggactgcttttttgtacaaacttgTTCGAGAAGAGAAGTCGAGAGAGA |  |
| fadA-3f | ggggacaactttgtatagaaaagttgttCTTGAAGCACAAGCTCGTTAAAT | For amplification of the *PlFadA* gene 3’ flank |
| fadA-3r | ggggacaactttgtataataaagttgtCACGCATGTTTGCAAATTATAGA |  |
| wetA-5f | ggggacagctttcttgtacaaagtggaaTGTACTTCGCACATTTTAGTTGC | For amplification of the *PlwetA* gene 5’ flank |
| wetA-5r | ggggactgcttttttgtacaaacttgtGTATTGGGGTTGGTGTTTGTTC |  |
| wetA-3f | ggggacaactttgtatagaaaagttgttCGCGTAACTGTGCTATATGTCCT | For amplification of the *PlwetA* gene 3’ flank |
| wetA-3r | ggggacaactttgtataataaagttgtGGATAGAATGCGTGAGAGGTTT |  |
| abaA-5f | ggggacagctttcttgtacaaagtggaaCTCTTACCTTGGATGTGTCC | For amplification of the *PlabaA* gene 5’ flank |
| abaA-5r | ggggactgcttttttgtacaaacttgtTCAAACACATGAAACAACGG |  |
| abaA-3f | ggggacaactttgtatagaaaagttgttAAATGTATGCTCTGAAGGGG | For amplification of the *PlabaA* gene 3’ flank |
| abaA-3r | ggggacaactttgtataataaagttgtCCAAGCAAGCATGAACTATG |  |
| abaA-PlKu80-F | CCTCGGAAGGGAACAGAAAGT | For amplification of the replaced *PlabaA* gene fragment |
| abaA-PlKu80-R | CCCACAAGGACACGCAAAA |  |
| wetA- PlKu80-F | TCACTCACCTTTGCCACCCTT | For amplification of the replaced *PlwetA* gene fragment |
| wetA-PlKu80-R | GTCATCATCGTGCATCATTAGC |  |
| pkaA- PlKu80-F | GGCTTGAGGTCTCGGTAAA | For amplification of the replaced *PlpkaA* gene fragment |
| pkaA-PlKu80-R | GCAGCAGGAGCAGATGAAC |  |
| fadA- PlKu80-F | CCCGAAAGTGAAGTGAGGT | For amplification of the replaced *PlfadA* gene fragment |
| fadA-PlKu80-R | TGGCTGCAAGGGTATGAG |  |
| PlWetA-C-F | CGAGGGATTCTGGCTTCTACTGG | Validation primers of *PlWetA* complement transformants |
| PlWetA-C-R | CGCGTGGCTGTTGAGATTGA |  |
| abaA-sb-f | GAGGAGGACGGGCAATG | For amplification of the Southern blot probe for *PlAbaA* |
| abaA-sb-r | TCGAGGCGCAATCTGTG |  |
| wetA-sb-f | CGGACATAAGATAGACCAACGAC | For amplification of the Southern blot probe for *PlWetA* |
| wetA-sb-r | GGGAAACGGGCAACCAG |  |
| fadA-sb-f | AGGCATTGCGACTCCAGG | For amplification of the Southern blot probe for *PlFadA* |
| fadA-sb-r | CGTCCATCTCAGCGACCAT |  |
| pkaA-sb-f | GCAGGTGCGTGCTTTCG | For amplification of the Southern blot probe for *PlPkaA* |
| pkaA-sb-r | GCTTTGAGGCTTGGTGGG |  |
| flbA-5f | ggggacagctttcttgtacaaagtggaaCCCACCGCATCACCATC | For amplification of the *PlflbA* gene 5’ flank |
| flbA-5r | ggggactgcttttttgtacaaacttgtGGATTCCGTGCCCAAGT |  |
| flbA-3f | ggggacaactttgtatagaaaagttgttGGTGGTAGTGGTGATGCTTTG | For amplification of the *PlflbA* gene 3’ flank |
| flbA-3r | ggggacaactttgtataataaagttgtTGGCGCACCGTTCAGTT |  |
| flbC-5f | ggggacagctttcttgtacaaagtggaaACCCGTGCCCAGTGAGA | For amplification of the *PlflbC* gene 5’ flank |
| flbC-5r | ggggactgcttttttgtacaaacttgtGTAATCGTACATATCCTTCGGGT |  |
| flbC-3f | ggggacaactttgtatagaaaagttgttAGCGTGTTTGTCGGAAGC | For amplification of the *PlflbC* gene 3’ flank |
| flbC-3r | ggggacaactttgtataataaagttgtGCATCAACCCATCTGGCT |  |
| flbD-5f | ggggacagctttcttgtacaaagtggaaTCATTGTCATTTGGTGGAGTC | For amplification of the *PlflbD*gene 5’ flank |
| flbD-5r | ggggactgcttttttgtacaaacttgtATACCATCCGACCGACAGA |  |
| flbD-3f | ggggacaactttgtatagaaaagttgttTAGGTGACTGGCTGAAGGAG | For amplification of the *PlflbD* gene 3’ flank |
| flbD-3r | ggggacaactttgtataataaagttgtTCAACACGCACCATTTCC |  |
| fluG-5f | ggggacagctttcttgtacaaagtggaaGCTCTTGCGAGGGAAAT | For amplification of the *PlfluG* gene 5’ flank |
| fluG-5r | ggggactgcttttttgtacaaacttgtGAACAACGACGACGATTATG |  |
| fluG-3f | ggggacaactttgtatagaaaagttgttTGCTGGGACTGTGGTAG | For amplification of the *PlfluG* gene 3’ flank |
| fluG-3r | ggggacaactttgtataataaagttgtGAAGGCAAAGGCTCATA |  |
| flbA-f | TCCAACATCCGCCTTCC | Validation primers of *PlflbA* knockouts |
| flbA-r | TCCATACCAAACCCGACAA |  |
| flbC-f | AAATGCTCCACTAACCAACAA | Validation primers of *PlflbC* knockouts |
| flbC-r | GGCTTCCGACAAACACG |  |
| flbD-f | GCCAGCCAGACTACAACAAA | Validation primers of *PlflbD* knockouts |
| flbD-r | GCACATACGCATACACTACCG |  |
| fluG-f | AACGACGACGATTATGTAG | Validation primers of *PlfluG* knockouts |
| fluG-r | AGGCTGGAGTTAGGGTAT |  |
| flbA-sb-f | AAAGGACGGCTGCTGCTC | For amplification of the Southern blot probe for *PlflbA* |
| flbA-sb-r | CCTCGCCTGTTGGAAAGC |  |
| flbC-sb-f | GGCAACGGGAGGAACAG | For amplification of the Southern blot probe for *PlflbC* |
| flbC-sb-r | CGGCAGACTACACGCACA |  |
| flbD-sb-f | CCATCGCCTGTTCGTCC | For amplification of the Southern blot probe for *PlflbD* |
| flbD-sb-r | CGTTGGAACTTGGCACTCTAT |  |
| fluG-sb-f | GAGGCAGTCAGATGTTG | For amplification of the Southern blot probe for *PlfluG* |
| fluG-sb-r | TAGAGTGTAGCAATAGC |  |
| RT-actin-F | GAGGTAGTCGGTCAAGTCGC | Real-time PCR for *Plactin* gene (reference gene) |
| RT-actin-R | TCCCATCAACCCCAAGTCC |  |
| RT-abaA-F | AGCTTGGGAGGAGTTGAGGA | Real-time PCR for *PlabaA* gene |
| RT-abaA-R | TTAGGCAGTACCGCAACAGG |  |
| RT-brlA-F | AGAAGGAAATGGGGCTGTCG | Real-time PCR for *PlbrlA* gene |
| RT-brlA-R | TTCCGGTCCCTATCCCATGT |  |
| RT-fadA-F | CGCGTGCGACTACATCCTTA | Real-time PCR for *PlfadA* gene |
| RT-fadA-R | TTCAAGAGCCAGCCAGTCAG |  |
| RT-flbA-F | GCTCCGTAACCTGCTTCTGT | Real-time PCR for *PlflbA* gene |
| RT-flbA-R | CTATTTGCCAGCGCTTCGTC |  |
| RT-flbC-F | GAAGCTCCTCCGGTCATCTG | Real-time PCR for *PlflbC* gene |
| RT-flbC-R | GTCGCTACTCCCATCACCAG |  |
| RT-fluG-F | AAGGGCGTCGTGGGCAATA | Real-time PCR for *PlfluG* gene |
| RT-fluG-R | CGGTCGTGGAGACATCGTG |  |
| RT-pkaA-F | TCCAGTCCTGAAATGTGCCC | Real-time PCR for *PlpkaA* gene |
| RT-pkaA-R | CTCAAGAAGGCCCAAGTCGT |  |
| RT-plbD-F | CCATCACCATCCTCGTCCTG | Real-time PCR for *PlplbD* gene |
| RT-plbD-R | CTGCCTCAACATCATCGGGT |  |
| RT-wetA-F | ACCTGTCCCAGCAATTCGAG | Real-time PCR for *PlwetA* gene |
| RT-wetA-R | GCGCCTCGACAAACTTCTTG |  |
| RT-TSL1-F | AGCCTTTTGTCTTTGTAGCG | Real-time PCR for *Pltsl1* gene |
| RT-TSL1-R | CTGTAGCCGTCTCCTTTCC |  |
| RT-TPS1-F | TGACCTCCTGGTAGGCAATC | Real-time PCR for *Pltps1* gene |
| RT-TPS1-R | CGCCACTATAACGGCTTCTC |  |
| RT-TPS2-F | GCGACGATTTCACCGACGAG | Real-time PCR for *Pltps2* gene |
| RT-TPS2-R | CGCCACGCAGTCAATCACG |  |
| RT-TPS3-F | TCAATGGCAACGAACTGT | Real-time PCR for *Pltps3* gene |
| RT-TPS3-R | TAAGAAGCTCCCCAAACC |  |
